# Peak emission wavelength and fluorescence lifetime are coupled in far-red, GFP-like fluorescent proteins

**DOI:** 10.1371/journal.pone.0208075

**Published:** 2018-11-28

**Authors:** Laura Canty, Santosh Hariharan, Qian Liu, Steven A. Haney, David W. Andrews

**Affiliations:** 1 Department of Biological Sciences, Sunnybrook Research Institute, Toronto, Ontario, Canada; 2 Department of Medical Biophysics, University of Toronto, Toronto, Ontario, Canada; 3 Department of Oncology and Translational Research, Eli Lilly and Company, Indianapolis, Indiana, United States of America; 4 Department of Biochemistry, University of Toronto, Toronto, Ontario, Canada; University of Campinas, BRAZIL

## Abstract

The discovery and use of fluorescent proteins revolutionized cell biology by allowing the visualization of proteins in living cells. Advances in fluorescent proteins, primarily through genetic engineering, have enabled more advanced analyses, including Förster resonance energy transfer (FRET) and fluorescence lifetime imaging microscopy (FLIM) and the development of genetically encoded fluorescent biosensors. These fluorescence protein-based sensors are highly effective in cells grown in monolayer cultures. However, it is often desirable to use more complex models including tissue explants, organoids, xenografts, and whole animals. These types of samples have poor light penetration owing to high scattering and absorption of light by tissue. Far-red light with a wavelength between 650-900nm is less prone to scatter, and absorption by tissues and can thus penetrate more deeply. Unfortunately, there are few fluorescent proteins in this region of the spectrum, and they have sub-optimal fluorescent properties including low brightness and short fluorescence lifetimes. Understanding the relationships between the amino-acid sequences of far-red fluorescence proteins and their photophysical properties including peak emission wavelengths and fluorescence lifetimes would be useful in the design of new fluorescence proteins for this region of the spectrum. We used both site-directed mutagenesis and gene-shuffling between mScarlet and mCardinal fluorescence proteins to create new variants and assess their properties systematically. We discovered that for far-red, GFP-like proteins the emission maxima and fluorescence lifetime have a strong inverse correlation.

## Introduction

Fluorescent proteins are powerful tools that enable the monitoring of gene expression, protein localization, and physiological processes in living cells [1–3]. Since the successful cloning of green fluorescent protein (GFP) [3], many efforts have been devoted to improving its fluorescent properties including increasing brightness and stability and expanding the color palette[4–14]. Fluorescent proteins with a red-shifted spectrum are of particular importance for two major reasons. First, they allow multiplexing with other fluorescent proteins in the blue, green, and orange region of the spectrum, enabling simultaneous monitoring of multiple proteins or processes. Second, far-red fluorescence proteins are advantageous for organoid, tissue, and animal imaging. Tissues absorb and scatter light in the visible region of the spectrum thus effectively limiting penetrance to a few microns. By comparison, light with wavelengths between 650–900 nm is better able to penetrate tissues because molecules such as water, oxygenated hemoglobin, and melanin absorb substantially less light in this region of the spectrum [15]. The discovery of other naturally occurring GFP-like proteins with red-shifted spectra, (e.g. red fluorescent protein from the coral Discosoma striada, dsRFP [16] and red fluorescent protein from the sea anemone Entacmaea quadrucolor, eqFP578 [13]) facilitated the expansion of fluorescent proteins into the red and far-red region of the spectrum. However, far-red fluorescent proteins tend to have a low quantum yield meaning they are dimmer, and they tend to have shorter fluorescence lifetimes than blue-shifted fluorescent proteins (Table 1), which reduces their efficacy for many applications including FRET-based assays.

**Table 1:** Properties of fluorescent proteins in the far-red region of the spectrum.

	λ_ex_^a^(nm)	λ_em_^b^(nm)	Molecular extinction coefficient (mM^-1^cm^-1^)	Quantum yield	Brightness^c^	Fluorescence lifetime (ns)		References
mScarlet	569	594	100	0.7	70		3.9	[17]
mKate2	588	633	62.5	0.4	25		2.5	[17,18]
mPlum	590	637	160	0.04	6.4		N.D.	[19]
mNeptune	600	650	67	0.2	13.4		N.D.	[20]
Turbo650	592	650	65	0.24	15.6		1.5^d^	[21]
TagRFP657	611	657	34	0.1	3.4		N.D.	[22]
mMaroon1	609	657	80	0.11	8.8		N.D.	[23]
mCardinal	604	659	87	0.19	16.5		1.3	[24]
NirFP	605	670	70	0.06	4.2		N.D.	[21]
mGarnet2	598	671	105	0.087	9.1		N.D.	[25]
TagRFP675	598	675	46	0.08	3.7		0.9	[26]
iRFP670	643	670	114	0.111	12.6		0.93	[27,28]
IFP1.4	684	708	92	0.077	7.1		N.D.	[27]
iRFP713	690	713	98	0.063	6.1		0.63	[27,28]

^a^. Peak exaction wavelength,

^b^. Peak emission wavelength,

^c^. Calculated as the product of extinction coefficient and quantum yield,

^d^. This study,

N.D. not determined

There is another class of fluorescent proteins that have emission spectra that fall well into the optical window of tissues (between 650 and 900 nm). These fluorescent proteins are derived from bacterial phytochrome receptors [29] and have a completely different structure than GFP and GFP-like proteins. Members of this class of fluorescent protein have peak emission wavelengths of 670–720 nm [30]. Similar to the far-red fluorescent proteins, these fluorescent proteins have relatively low quantum yields and fluorescence lifetimes between 0.5 and 1 ns (Table 1; [28]). However, for use as acceptors the fluorescence lifetime is not critical and these near-infrared fluorescent proteins are of the appropriate wavelength for use as acceptor proteins for far-red fluorescence proteins in FRET-based biosensors [31,32].

One of the brightest GFP-type fluorescent proteins with a peak emission beyond 650 nm is mCardinal, with excitation and emission maxima at 604nm and 659nm respectively [24] and a brightness, calculated by taking the product of the molecular extinction coefficient and quantum yield, of 16.5. However, compared to mScarlet with an emission maximum of 594 nm, a brightness of 70 and a fluorescence lifetime of 3.9 ns, mCardinal is much dimmer and has a relatively short fluorescence lifetime of 1.3 ns [24]. To date, it is not clear to what extent these properties are linked and what limits the fluorescence lifetime of far-red fluorescence proteins.

Fluorescence lifetime is the average amount of time the donor remains in the excited state before releasing a photon and is characteristic of each fluorophore. Fluorescence lifetime is particularly useful for measuring FRET in live cells and can be used with genetically encoded fluorescent biosensors. FRET results in a decrease in fluorescence lifetime of the donor fluorophore and emission of a photon from the acceptor rather than the donor fluorophore [33]. Both lifetime and intensity measurements have been used to evaluate FRET.

Fluorescence lifetime is a powerful way to measure FRET because it is independent of excitation intensity and fluorophore concentration making quantitative measurements in live cells practical even when the two fluorophores are expressed as separate proteins rather than linked in a single protein [34]. Because the signals are noisy and there can be both bound and unbound proteins in a single pixel it is important for the donor to have as close to a monoexponential decay as possible. Furthermore, an extended fluorescence lifetime of the donor fluorophore results in a larger dynamic range assay [34,35]. For example, mClover, with a fluorescence lifetime of 3.2 ns has been found to be a superior donor for use in FLIM-based assays because it results in FLIM measurements with a better dynamic range relative enhanced GFP (EGFP), with a fluorescent lifetime of only 2.4 ns [35]. In practice a donor with a fluorescence lifetime greater than 3ns is required to distinguish random collisions between colocalized molecules and bona fide protein binding. Therefore, to have optimized FRET-based biosensors for use in FLIM-based assays, a donor with a fluorescence lifetime of at least 3.0 ns is highly desirable. While most fluorescent proteins have a fluorescence lifetime between 0.1 and 4.0 ns [36], the few fluorescent proteins in the far-red region of the spectrum have short fluorescence lifetimes (< 1.5 ns) making them poor candidates for donor fluorophores in FRET-based biosensors for applications using FLIM.

Despite numerous successful improvements in the photophysical properties of fluorescence proteins our understanding of how to systematically modify fluorescent proteins to optimize specific fluorescent properties [13] remains rudimentary. For example, it is known that quantum yield and fluorescence lifetime are highly correlated [36] and rigidifying the chromophore is thought to increases the quantum yield, as well as increasing the fluorescence lifetime [36]. A rigid chromophore tends to improve quantum yield, brightness, and fluorescence lifetime because it reduces non-radiative energy loss resulting from vibrations, rotations, and other conformational changes [36]. These non-radiative means of energy loss are a form of intramolecular quenching which results in decreased brightness and fluorescence lifetime [36]. Thus, preventing non-radiative energy loss results in brighter fluorescent proteins with higher quantum yields and extended fluorescence lifetimes.

Many factors can affect the brightness of a fluorescent protein when it is expressed in cells. These include how well the fluorophore is able to absorb a photon, (the extinction coefficient), the probability that the a photon will be released once the fluorophore is in the excited state (quantum yield), and concentration of the fluorophore, which can be affected by the folding efficiency and stability of the protein. Although increased quantum yield and brightness are highly desirable characteristics they are complicated and time-consuming measurement not compatible with screening large numbers of mutants. Furthermore, fluorescent proteins that appear to be brighter in cells do not necessarily have high quantum yields. One example of this is mScarlet and mScarlet-I. mScarlet does not appear as bright in cells relative to mScarlet-I (313 and 363% brighter than mCherry respectively [17]). However, mScarlet has a higher quantum yield compared to mScarlet-I (0.70 and 0.54, respectively). The apparent difference between relative brightness in cells and the quantum yield is due to the difference in accumulation of the fluorescent proteins resulting from differences in the maturation times. mScarlet-I has an apparent delay in maturation relative to mTurquoise of 0.6 hours and an accumulation of 129% in cells normalized to mCherry, while mScarlet has an apparent delay in maturation relative to mTurquoise of 2.9 hours and an accumulation of 89% in cells normalized to mCherry [17]. The Fluorescence lifetimes for these proteins correlate with the quantum yield as mScarlet has a longer fluorescence lifetime relative to mScarlet-I (3.9 ns and 3.1 ns, respectively [17]). Therefore, we measured fluorescence lifetime as the primary read-out from our assays.

To date, the brightest red fluorescent protein, with the most extended fluorescence lifetime (3.9 ns) is mScarlet [17]. While mScarlet performs well in terms of fluorescence lifetime, it is not ideal for applications in tissue or whole animal imaging because its excitation and emission peaks, at 569 nm and 594nm respectively [17], fall short of the optical window of tissues. However, because mScarlet is unique in its brightness and fluorescence lifetime among other fluorescent proteins in the red to far-red region of the spectrum, it may provide insight into the requirements necessary to improve the brightness in other far-red fluorescent proteins. Other fluorescent proteins in this region of the spectrum may be less informative in this context because they generally do not perform very well.

To better understand the relationship between emission maximum and lifetime of far-red fluorescence proteins we used site-directed mutagenesis of mCardinal and gene shuffling between mCardinal and mScarlet [37,38]. Our rationale for the gene shuffling was to mix two existing fluorescent proteins, one with a long lifetime but shorter emission maximum and the other with a short lifetime but longer emission maximum to find a fluorescent protein with both properties optimized. However, we discovered that there is a strong negative correlation between fluorescence lifetime and emission maximum suggesting there is a spectral limit to what can be achieved using GFP-like proteins.

## Methods and materials

### Tissue culture

HEK293T cells were used to test the point mutations because the ultimate goal was to use the optimized fluorescent protein in mammalian cells. HEK293T are good model cell line to apply for this purpose because they are particularly easy to grow and transfect. The HEK293T cells [39] were a gift from Frank Graham at McMaster University. Cells were grown in DMEM complete media. Cells were transiently transfected using calcium phosphate [40] for 16 hours. Cells were allowed to grow for another 24 hours after media change before imaging or harvesting. For spectral analysis experiments, cells were grown and transfected in 10 cm dishes. 24 hours after transfection cells were washed three times with PBS and once with hypotonic lysis buffer (1.5 mM MgCl_2_, 100nM HEPES, pH = 7.4, and protease inhibitors). Cells were incubated in 1 mL hypotonic lysis buffer on ice for 10 mins before homogenization using a Potter homogenizer. The suspension was centrifuged at 200 g for 10 mins at 4°C, and the supernatant was used for spectral analysis on a PTI QuantaMaster Fluorimeter from Horiba using FelixGX software (v. 4).

### Mutants

All mCardinal and mKate2 mutants were synthesized by DNA2.0 (Now ATUM; Newark, California) in mammalian expression vectors under a CMV promoter. The mScarlet/mCardinal shuffle library was created by ATUM with products cloned into the pD144-SR expression vector with an IPTG inducible T5 promoter.

### Fluorescence lifetime imaging microscopy (FLIM)

Cells were seeded in Cell Carrier 384-Ultra plates (6057308, Perkin Elmer) for imaging. Fluorescence lifetime images were taken as described [41]. Briefly, we used an ISS-Alba FLIM/FCS confocal microscope with a 60x water immersion objective (NA = 1.2) and a PDL 800-D pulsed laser (PicoQuant) at a repetition rate of 20MHz. We used a 445/505/588nm excitation filter and a 628/30 nm emission filter. VistaVision Software was used for acquisition and analysis of the FLIM data.

### The gene–shuffle screen

The gene-shuffle library was screened in bacteria because it is faster and easier to screen a large number of colonies and recover the DNA of potential hits in the library in bacteria than it would be using a mammalian cell line. The mScarlet/mCardinal shuffle library was randomly generated as a pooled library [30] by ATUM (Newark, California) in an E. coli expression vector under an IPTG inducible T5 promoter. The library was transformed into T7 shuffle E. coli (C3029, NEB). All colonies arising from the transformation were selected and cultured overnight at 37°C in 96-well Megablocks (83.1972.002, Sarstedt) in the presences of ITPG (IPT002.50, Bioshop). 100 μL of bacterial suspension was transferred to non-binding, 96-well half area assay plates (3881, Corning) for emission scans. Fluorescent emission was scanned from 620–750 nm on a TECAN infinity M1000pro fluorescence plate reader with excitation at 600 nm. Plasmid DNA was recovered and sequenced for all bacteria expressing proteins with fluorescent intensity > 1000 A.U. between 620 and 750 nm with excitation at 600 nm (very close to the excitation maximum of mCardinal at 604 nm). These mutants were also analyzed for fluorescence lifetime, and full excitation and emission scans were recorded.

### Excitation and emission scans

Excitation and emission scans were performed on PTI QuantaMaster Fluorimeter from Horiba using FelixGX software (v. 4). 300 μL of cell lysate or bacterial suspension were placed in special optical glass cuvettes with 4 mm path length (23–4.45-SOG-4, Starna Cell, Inc.). Measurements were taken at 25°C. Measurements were taken at 1 nm intervals with a 1 second integration time.

### Lifetime measurement

Decay curves were obtained on a PTI QuantaMaster Fluorimeter from Horiba using FelixGX software (v. 4) and a whitelase SC450 pulsed laser using a repetition rate of 20MHz. 300 μL of cell lysate or bacterial suspension were placed in special optical glass cuvettes with 4mm path length (23–4.45-SOG-4, Starna Cell, Inc.). Measurements were taken at 25°C until peak photon count reached 10,000. Lifetimes were calculated from the decay cures using the data analysis package from FelixGX. All fluorescence lifetimes were calculated by fitting a mono-exponential decay. Selected decay curves are shown in (S3 Fig).

### Random forest regression

We performed a Random Forest regression [42] to determine residues that are important for determining emission wavelength or fluorescence lifetime. The individual positions were used as features while the amino acid in each position was used as a categorical variable. Importance values were scaled as a proportion of the maximum value. For this analysis, we used only the protein sequences generated from the gene-shuffled library. We aligned the sequences of all the unique fluorescent proteins and used only the positions where the amino acids in mCardinal and mScarlet were different. Because this library was generated via gene shuffling, most loci had one of two amino acids (either the amino acid from mCardinal or the amino acid from mScarlet); however, there were a few loci that also included novel amino acids (i.e. not found in mCardinal or mScarlet). This appears to be a result of a mid-codon shuffling event resulting in a third amino acid (S1 Table).

Many of the residues that differ between mCardinal and mScarlet are in unstructured loop regions or on β-strands with their sidechains facing outwards and thus away from the chromophore making it difficult to predict how they might affect the fluorescent properties of the fluorescence protein (S4 Fig). However, it is possible that these residues are important for structural aspects of the protein, including overall rigidity that could potentially impact the fluorescent protein. Thus, we included all residues that differed between mCardinal and mScarlet in the analysis. For measuring the model performance, we used standard leave one out analyses where, in every run, one sequence was left out for validation while the rest of the sequences were used for generating the regression model.

## Results and discussion

### Site-directed mutagenesis of mCardinal

**Fig 1:**
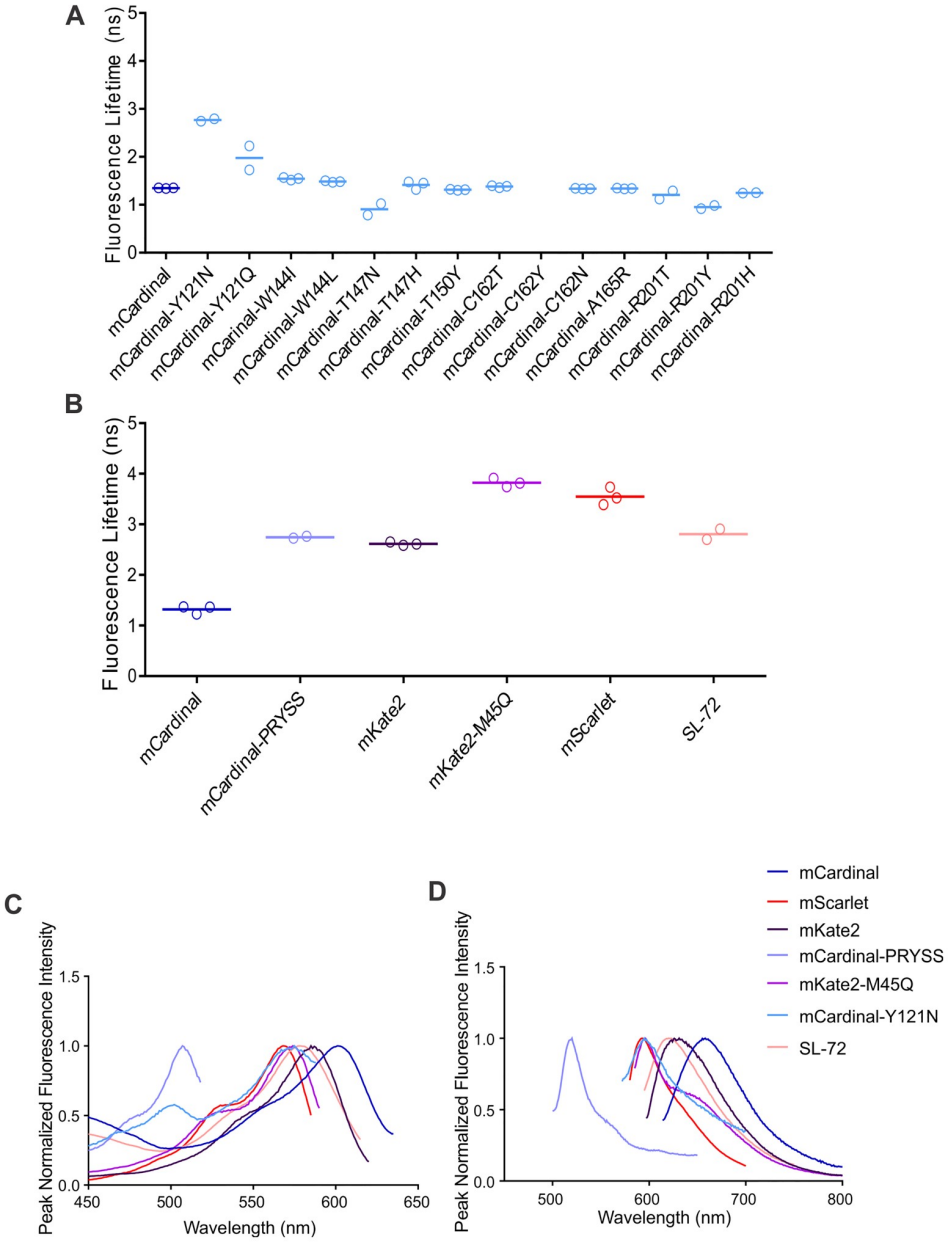
**A** Fluorescence lifetime measurements of mutants from site-directed mutagenesis of mCardinal measured in HEK393T cells by FLIM. **B** Fluorescence lifetime measurement of mutants from site-directed mutagenesis of mCardinal and mKate2 (in HEK293T cell lysates) and SL72 from the mScarlet/mCardinal shuffle library (in bacterial suspension) measured on the PTI fluorimeter. **C** Excitation and **D** Emission spectra of fluorescence proteins with the longest fluorescence lifetimes in HEK293T cell lysates, made by site-directed mutagenesis and SL72 from the mCardinal/mScarlet Shuffle library (measured in bacterial suspension).

We made 14 single site-directed mutants of mCardinal aimed at extending the fluorescence lifetime by modifying residues that were close to the chromophore and likely to impact fluorescence lifetime (S1 Fig). We based these mutations on mutations that had been shown to increase fluorescence lifetime in other fluorescent proteins, by comparing the amino acid sequence between mCardinal and other fluorescent proteins with longer fluorescence lifetimes and selecting residues expected to stabilize the chromophore or facilitate hydrogen bonding with the chromophore. Of these mutants, nine (mCardinal-W144I, W144L, T147H, T150Y, C162T, C162N, A165R, R201T, and 201H) had no effect on fluorescence lifetime, two of the mutants, mCardinal-T147N and mCardinal-R201Y reduced the fluorescence lifetime to 0.9 ns and 0.95 ns respectively while one mutant, mCardinal-C162Y, was no longer fluorescent (Fig 1A). Two mutants, mCardinal-Y121N, and mCardinal-Y121Q increased the fluorescence lifetime to 2.7 ns and 2 ns, respectively (Fig 1A). However, these mutants also had a 64 nm blue-shift in their spectra (Fig 1C, S2 Fig).

The above mutagenesis was based on insights from fluorescent proteins including mCerulean3 [4] and mTurquoise [43], which are substantially blue-shifted relative to mCardinal. In an attempt to minimize the blue-shift, we focused on fluorescent proteins with extended fluorescence lifetimes that that were closer in the spectrum to mCardinal.

mScarlet is a very bright, red fluorescence protein with peak excitation and emission wavelength at 569 and 594 nm and fluorescence lifetime of 3.9 ns [17]. Based on the residues hypothesized by Bindels et al., to be important for the extended lifetime in mScarlet, we made the mCardinal-PRYSS (T64**P**, K71**R**, F84**Y**, T147**S**, A221**S**) mutant. While this mutant did have an extended lifetime (2.7 ns; Fig 1B) relative to wildtype mCardinal, it had a 139 nm blue-shift in the emission spectrum (Fig 1C; S2 Fig).

### Site-directed mutagenesis of mKate2

Kate2 has a peak excitation and emission wavelengths of 588 and 633 nm [44] and a fluorescence lifetime of 2.5 ns [17], as well as high sequence similarity to mCardinal [44] meaning that there are very few residues that could potentially account for the difference in fluorescence lifetime. Of the residues that differ, residue 45 (S1 Fig) stood out as being potentially important for at least one of these properties because it is close to the chromophore and there is a polar residue (glutamine) in mCardinal versus a hydrophobic residue (methionine) in mKate2. We hypothesized that a change in hydrophobicity close to the chromophore could be responsible for the differences in spectra and/or fluorescent lifetime found between mKate2 and mCardinal. Thus, we mutated the methionine at residue 45 in mKate2 to glutamine (mKate2-M45Q) as found in mCardinal at this position to see the effect this residue has on the emission maximum and/or fluorescent lifetime. Surprisingly, this mutation resulted in a 35 nm blue-shift relative to wildtype mKate2 (Fig 1C; S2 Fig) as well as an increase in fluorescence lifetime to 3.8 ns (Fig 1B).

Together with the data from the site-directed mutagenesis in mCardinal, our results suggest that we cannot easily apply the same mutations that resulted in an increase in fluorescence lifetime for other fluorescent proteins to fluorescent proteins in the far-red region on the spectrum. Even though the structures are very similar across the GFP-like proteins, the primary sequence can differ substantially, and specific mutations may not have the same outcome in a different environmental context created by a difference in primary sequence elsewhere in the protein.

One mechanism that could explain the blue-shift in the spectrum is that the mutations act to increase the energy gap between the ground state and the excited state of the chromophore. This would be likely in mutations that resulted in a moderate blue-shift in the spectrum. For example, the methionine at position 45 in mKate2 and the tyrosine at position 121 in mCardinal are both very close to the chromophore (S1 Fig) and could interact with it directly, thus mutating these residues to glutamine and asparagine, respectively, could alter the energy required reach the excited state and thus account for the 38 and 64 nm blue-shifts in the spectrum of mKate2-M45Q and mCardinal-Y121N mutants resulting from a single, non-conservative amino acid substitution.

The structure the chromophore can greatly impact the spectral properties of the fluorescent protein [45]. The mCardinal-PRYSS mutant is highly blue shifted but this is almost certainly due to a change in chromophore structure. That is, this variant forms a GFP-type chromophore rather than a DsRed-type chromophore resulting in the extreme blue-shift in the spectrum of 139 nm. Thus, another possible explanation for the moderate to large blue-shifts observed in the mutants is that some of the mutations we introduced affected the maturation of the chromophore resulting in GFP-like chromophore structures rather than the DsRed structure. Further, as described in Bindels et al., 2017, many residues in different regions of the protein were thought to be necessary for the extended fluorescence lifetime seen in mScarlet. The same could also be true for residues responsible for the spectral properties of other far-red fluorescent proteins. For these reasons, we moved away from a site-directed approach and towards a random approach.

### Gene-shuffling between mCardinal and mScarlet

We screened a library of fluorescent proteins generated via gene-shuffling between mScarlet and mCardinal. We performed gene-shuffling because it has been proposed that this method covers a larger phenotypic distribution versus random mutagenesis of mCardinal or mScarlet alone [38]. We chose to shuffle mScarlet and mCardinal because mScarlet has the longest fluorescence lifetime of the red fluorescent proteins, and mCardinal has an emission maximum that falls within the optical window while still being relatively bright. Of approximately 10,000 proteins screened from the gene-shuffle library, many were not fluorescent when excited with light at 600 nm to select for far-red fluorescent proteins. We selected all the proteins that had a fluorescence intensity of at least 1000 A.U. at some wavelength between 620 and 700nm for further analysis. From this subset of proteins, we identified both mScarlet and mCardinal, along with 81 unique fluorescent proteins with peak emission wavelengths and fluorescent lifetimes that fell between mScarlet and mCardinal (S1 Table). The most red-shifted protein with fluorescence lifetime approaching 3.0 ns (SL72 highlighted in S1 Table) has a peak excitation and emission wavelengths of 578 and 621 nm (Fig 1C; S2 Fig) and a fluorescence lifetime of 2.8 ns (Fig 1B). However, this protein is not as red-shifted as mKate2 with excitation and emission maxima at 588 and 633 nm and has only a very slight increase in fluorescence lifetime from 2.5 ns for mKate2 to 2.8 ns for SL72.

**Fig 2:**
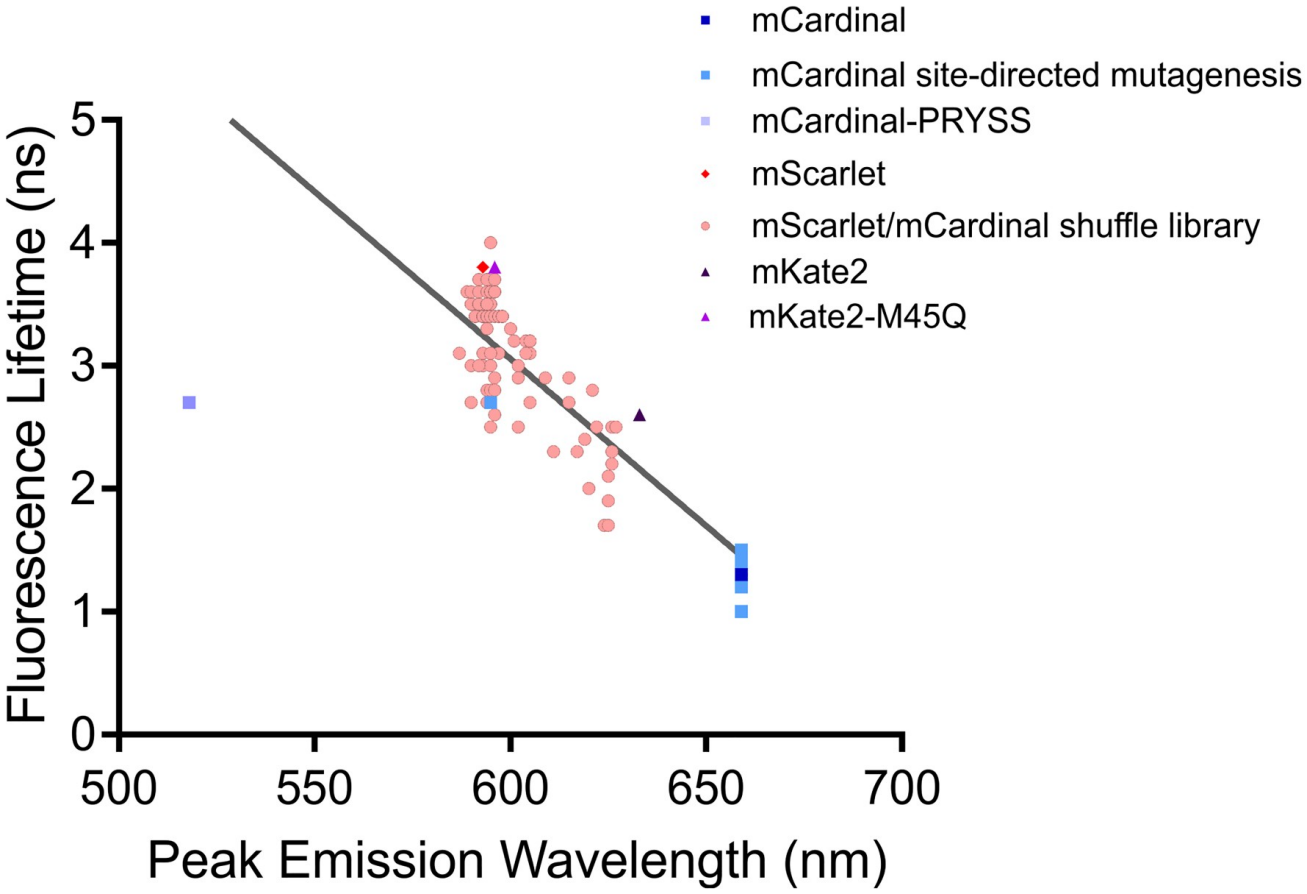
Fluorescence lifetime plotted against peak emission wavelength for all unique fluorescent proteins generated either by site-directed mutagenesis or from the mScarlet/mCardinal shuffle library.

Interestingly, when we plotted peak emission wavelength against fluorescence lifetime (Fig 2), we observed a strong and significant (p < 0.0001) negative correlation (R^2^ = 0.6998) between the peak emission wavelength of the fluorescent proteins and the fluorescent lifetime suggesting that peak emission wavelength and fluorescence lifetime cannot be independently manipulated. Another surprising finding was that we did not find any new fluorescent proteins with peak emission wavelength greater than 625 nm (pink circles in Fig 2; S1 Table). We had expected a continuous distribution of peak emission wavelength between mScarlet (594 nm) and mCardinal (659 nm). The fact that we did not observe any novel proteins with peak emission wavelengths beyond 625 nm suggests that we are approaching the spectral limit of this fluorophore. While it is possible that there were fluorescent proteins in the gene-shuffle library with peak emission wavelengths beyond 659 nm that did not meet our fluorescence intensity cut-off of 1000 A.U., this still represents a potential limit in the spectrum beyond which the fluorescence is not bright enough to be useful. Thus, a very specific set of properties at particular residues are likely required to have a detectable fluorescence emission maximum beyond 625 nm. We speculate that even minor changes to these residues could result in a blue-shift in the spectrum or complete loss of fluorescence. This hypothesis would also explain the unexpected results we obtained from the site-directed mutagenesis we performed.

### Random forest regression

**Fig 3:**
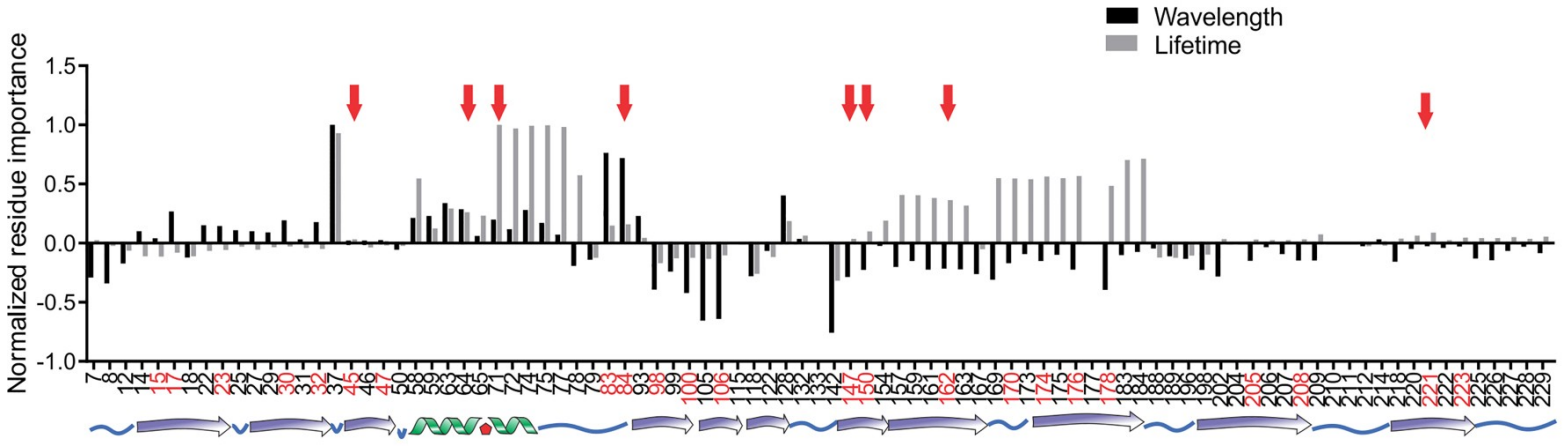
Residue importance for determining fluorescence lifetime (grey bars) or on peak emission wavelength (black bars) scaled as a proportion of the maximum importance for each property. Positive numbers indicate that the residue is of relative importance and negative values mean the residue is not important. Numbers on the x-axis correspond to residue number in mCardinal. The secondary structure of the protein is illustrated along the bottom of the graph. Residues 58–79 make up the alpha helix and loop that hold the chromophore (Red pentagon) inside the β-barrel. Numbers highlighted in red indicated those residues on the β-sheet or loop regions that face inside the β-barrel and thus, could interact with the chromophore. Red arrows indicate residues that were independently selected for the site-directed mutagenesis in mCardinal or mKate2.

To address these hypotheses further, we used random forest regression [42] using the gene-shuffling data to test the null hypothesis and identify regions of the protein that might be important for wavelength or fluorescence lifetime separately. This analysis predicts regions of the protein that are important but has no directionality (Fig 3). Reassuringly, there were residues that were predicted by the model that were also independently identified by Bindels et al., 2017, and experimentally determined by us as being important for fluorescence lifetime. However, the overall conclusion from this analysis is that most regions predicted to be important for fluorescence lifetime were also predicted to be important for emission wavelength.

#### Residues important for fluorescence lifetime

Residues 157–184 were identified in our model as candidates for being important for only fluorescence lifetime. Interestingly, these residues make up two adjacent β-sheets. Unfortunately, only three residues in this region had been independently predicted to be important for fluorescence lifetime either by our group or Bindels et al. [17]. Residue 178 was identified by Bindels et al. [17] and we independently selected residues T150, and C162 for mutagenesis in mCardinal (Fig 3). Unfortunately, the point mutations, T150Y, C162N, and C162T in mCardinal did not result in a change in fluorescence lifetime, and the C162Y mutation in mCardinal resulted in a complete loss of fluorescence (Fig 1A). However, these results do not mean the model was wrong. Given that this model was based on the mScarlet/mCardinal shuffle data and these residues were mutated to amino acids that are not found in mScarlet or mCardinal at these positions it is possible that we did not select the correct amino acids to elicit a change in fluorescence lifetime. However, our results suggest that if further mutagenesis of this area were carried out we would likely only find amino acids with negative effects on fluorescent properties and so it was not pursued further.

#### Residues important for emission spectra

There was only one region that was predicted as being important only for determining the emission maxima. That sequence included residues 22–32 close to the N-terminus of the protein with a relatively low probability of affecting emission wavelength. In our screen, we only found two novel fluorescent proteins had the amino acids from mCardinal in this region, and they both had emission maxima above 605 nm (S1 Table). However, there were many other fluorescent proteins with emission maxima 605 nm and greater, which suggested there may be other regions in the protein that are important for red-shifting the spectrum, including residues at positions 83 and 84, although, these residues are also predicted to be important for fluorescence lifetime. Thus, our data and model suggest that with the chromophore in these fluorescence proteins it may not be possible to have a far-red fluorescent protein with an extended fluorescence lifetime.

Notably, none of the novel fluorescent protein from the gene-shuffle library had an emission maximum that came close to mCardinal, and very few fluorescent proteins had residues from mCardinal in the N-terminal region particularly from residue 22–51, which includes the region identified by the model as being important for emission. To see if this entire region could account for the extra red-shift seen in mCardinal, we replaced the residues 22–51 in SL72 with the residues found in mCardinal and found that this mutation resulted in a complete loss of fluorescence (data not shown). This data supports the hypothesis that we are hitting the spectral limit of this fluorescent protein and it suggests that it is only possible to achieve the emission maximum seen in mCardinal under a specific context dependent on the amino acids present across multiple regions of the protein.

#### Residues important for both emission peak and fluorescence lifetime

Some of the remaining residues were predicted to be important for both the emission spectra and for fluorescence lifetime. One region of note was from residue 71 to residue 77. These residues make the α-helix that holds the chromophore inside the β-barrel. Because of their proximity to the chromophore, it is not surprising that this region is important in determining the spectral and photophysical properties such as fluorescence lifetime.

Three of the residues in this region had been predicted by Bindels et al., as being important for the extended fluorescence lifetime of mScarlet [17]. As mentioned previously, we mutated all three of these amino acids in mCardinal along with two other residues that were identified as being important for the extended lifetime of mScarlet to make the mCardinal-PRYSS mutant (S1 Fig). This mutant had an increase in fluorescence lifetime to 2.7 ns but also had a 64 nm blue-shift in the spectrum–resulting in a protein even more blue-shifted than mScarlet (Fig 1B and 1C, S2 Fig). These results support the prediction by the model that this region is important for both fluorescence lifetime and spectral properties.

The reason far-red fluorescent proteins have short fluorescence lifetimes is most likely related to the relatively small energy gap between the excited and the ground state. As stated previously, mutations near the chromophore may result in an increase in the energy gap between the excited state and the ground state, resulting in the smaller blue-shifts in the spectrum. This mechanism could explain the observation that the mutations we tested that successfully increased the lifetime also resulted in a blue-shift in the spectrum.

Alternatively, it is also possible that the some of the spectral properties in fluorescent proteins resulting from the shuffle library resulted from a change in the structure of the chromophore from the DsRed structure to GFP-like structure. Consistent with this hypothesis there are eight amino acids (residues 17, 45, 64, 65, 71, 147, 162, and 174 mapped to mCardinal; S1 Table residues with an asterisk) that differ between mScarlet and mCardinal and are very close to the chromophore (S4 Fig). These residues, when mutated, are the most likely candidates to elicit spectral changes due to alteration of the chromophore structure. Furthermore, none of the novel fluorescent proteins in the shuffle library contain the residues from mCardinal at all eight of the positions that are near the chromophore and listed above. This could suggest the structure of the DsRed chromophore is highly dependent on the surrounding amino acids and changes to some of these key amino acids may result in GFP-like structure rather than the DsRed structure, which is important for the extreme red-shift seen in mCardinal. However, further studies are needed to fully test this hypothesis and investigate the extent to which these mutations are affecting the structure of the chromophore.

## Conclusions

Our results suggest that we have identified the spectral limit of the chromophore in current far-red fluorescence proteins. As a result, amino acid sequences that result in peak emission wavelengths beyond 625 nm will be rare and will have short fluorescence lifetimes. We also hypothesize that very red-shifted fluorescent proteins, like mKate2 and mCardinal, are inherently more sensitive to spectral changes when mutations aimed at improving brightness or fluorescence lifetime are introduced even though similar mutations have been shown to have no impact on emission in more blue-shifted fluorescent proteins[4,17,44]. We, therefore, conclude that for FRET-based sensors mScarlet or mKate2 are the proteins of choice even though their emission maxima are not sufficiently red-shifted for use in animals. However, we predict that they will have reasonable properties for use in 3D tissue cultures. For imaging studies in which only localization is desired it will be important to counterbalance the further red emission offered by mCardinal against the much brighter mScarlet.

## Supporting information

S1 Fig Structural representation of mCardinal or mKate2 depicted using PyMOL [46] to visualize location of residues mutated in the site-directed mutagenesis (A) in the mCardianal-PRYSS mutant (B) shown in mCardinal (PDB ID: 4OQW) and M45 in mKate2 (C) shown on mKate (PDB ID: 4OQW). Note that the residues that are on the inner α-Helix are not shown for **A** and **C**, and a couple of β-strands have been removed from **B** for better visualization of the highlighted residues.
(TIF)

S2 Fig Peak-normalized excitation and emission spectra of the mCardinal (A), mCardinal-Y121N(B), mCardinal-PRYSS(C), mKate2 (D), mKate2-M54Q (E), mScarlet (F), and SL72 from the mScarlet/mCardinal shuffle library(G). (TIF)

S3 Fig Example decay curves of select fluorescent proteins, either expressed in cell lysates (mCardinal and mKate2 mutants) or E. coli (mScarlet and SL72), taken on the PTI. (TIF)

S4 Fig Structural representation of mCardinal with amino acids that differ from mScarlet depicted using PyMOL [46]. All amino acids that are on the β-strands that facing outward away from the chromophore (**A**), inward toward the chromophore (**B**), or the nine closest inward facing amino acids (**C**). All residues are shown on mCardinal (PDB ID: 4OQW). Note that the residues that are on the inner α-Helix are not shown. **D** The two amino acids that are from mCardinal and close to the chromophore in SL72. These residues are shown on mScarlet (PDB ID: 5LK4) with the mCardinal residues highlighted because SL72 is more similar to mScarlet than mCardinal.
(TIF)

S1 Table Amino acid sequence alignment of unique sequences from the mScarlet/mCardinal shuffle library with their respective excitation/emission maxima and fluorescence lifetime. Residue number is based on the mCardinal sequence and is indicated at the top of the chart with green numbers indicating residues that make up the inner α-helix, red numbers being residues in the β-barrel that point inward towards the chromophore and black numbers are residues in the β-barrel that point outward and away from the chromophore. Numbers with an asterisk are those residues whose sidechain is close to the chromophore and could potentially interact directly. Only residues that are different between mCardinal and mScarlet are shown. Those residues that are the same as mCardinal are highlighted in blue, and those residues that are in mScarlet are highlighted in Red. Residues that are not found in mCardinal or mScarlet are not highlighted (white background).
(XLSX)
