# Supplementary figures and images for: Peak emission wavelength and fluorescence lifetime are coupled in far-red, GFP-like fluorescent proteins
Source: PLoS One. 2018 Nov 28;13(11):e0208075. doi: 10.1371/journal.pone.0208075 (PMC6261627; doi:10.1371/journal.pone.0208075)

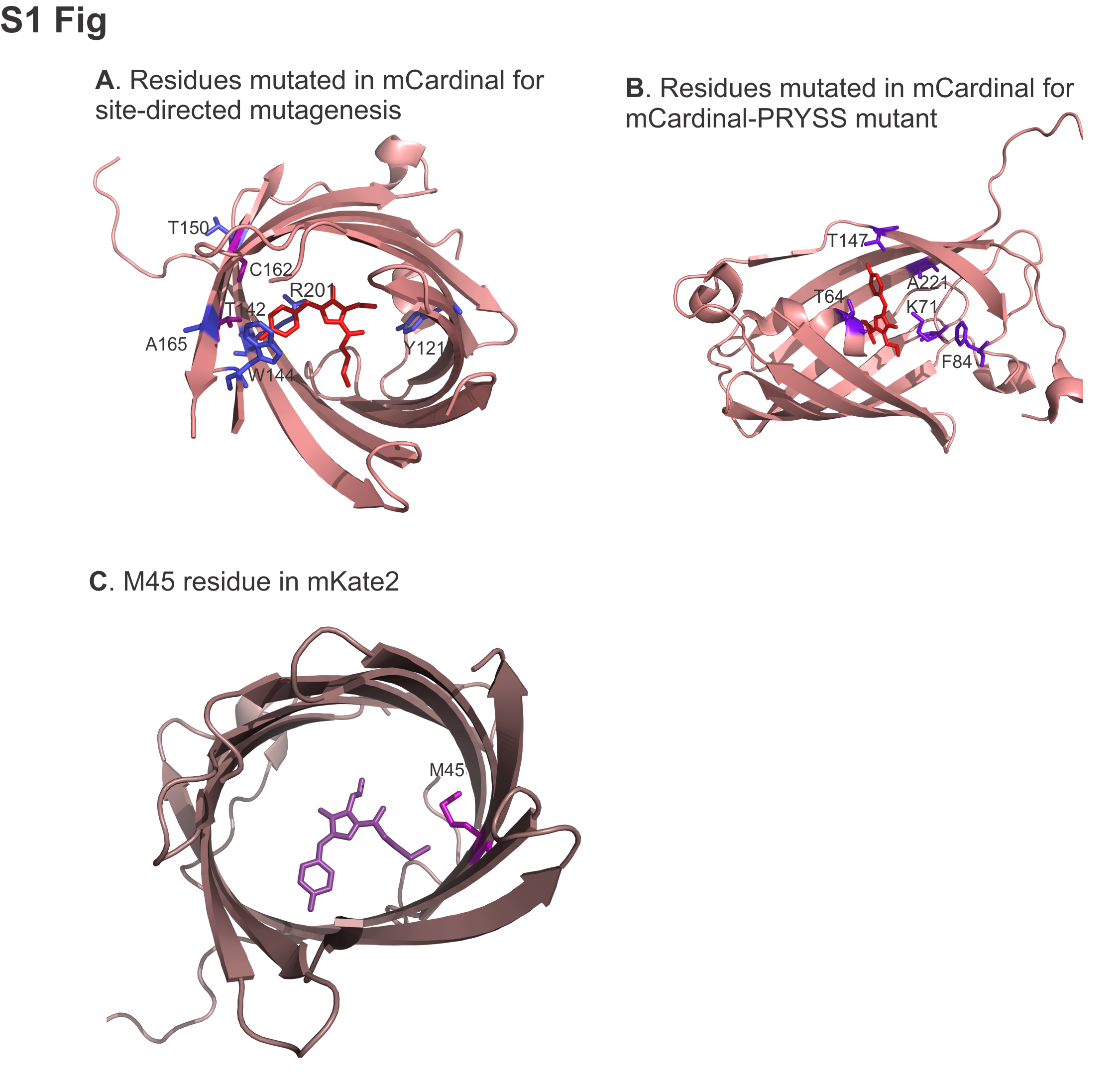

Supplement: S1 Fig — Note that the residues that are on the inner α-Helix are not shown for A and C, and a couple of β-strands have been removed from B for better visualization of the highlighted residues. (TIF) [file pone.0208075.s001.tif]

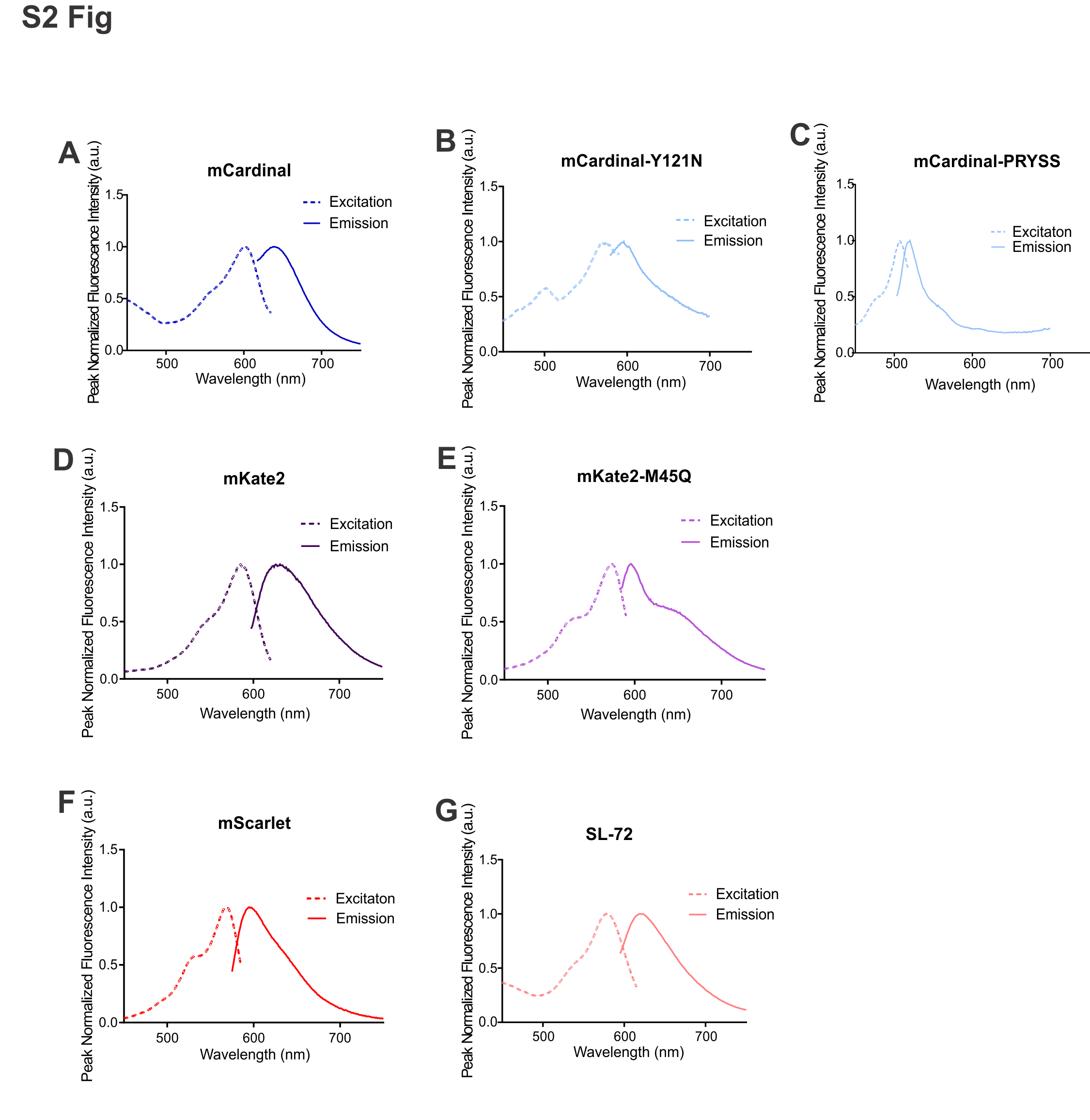

Supplement: S2 Fig — (TIF) [file pone.0208075.s002.tif]

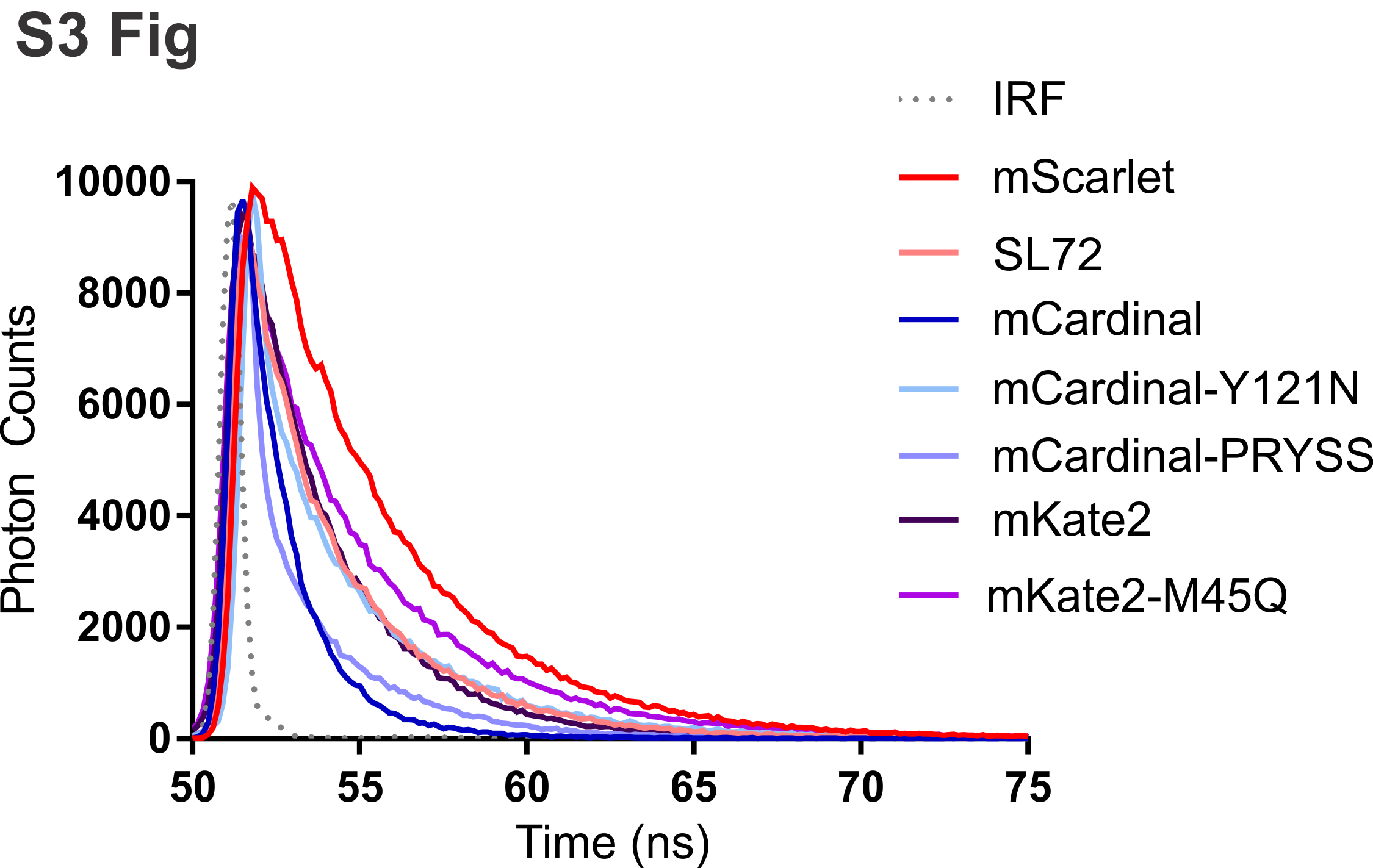

Supplement: S3 Fig — (TIF) [file pone.0208075.s003.tif]

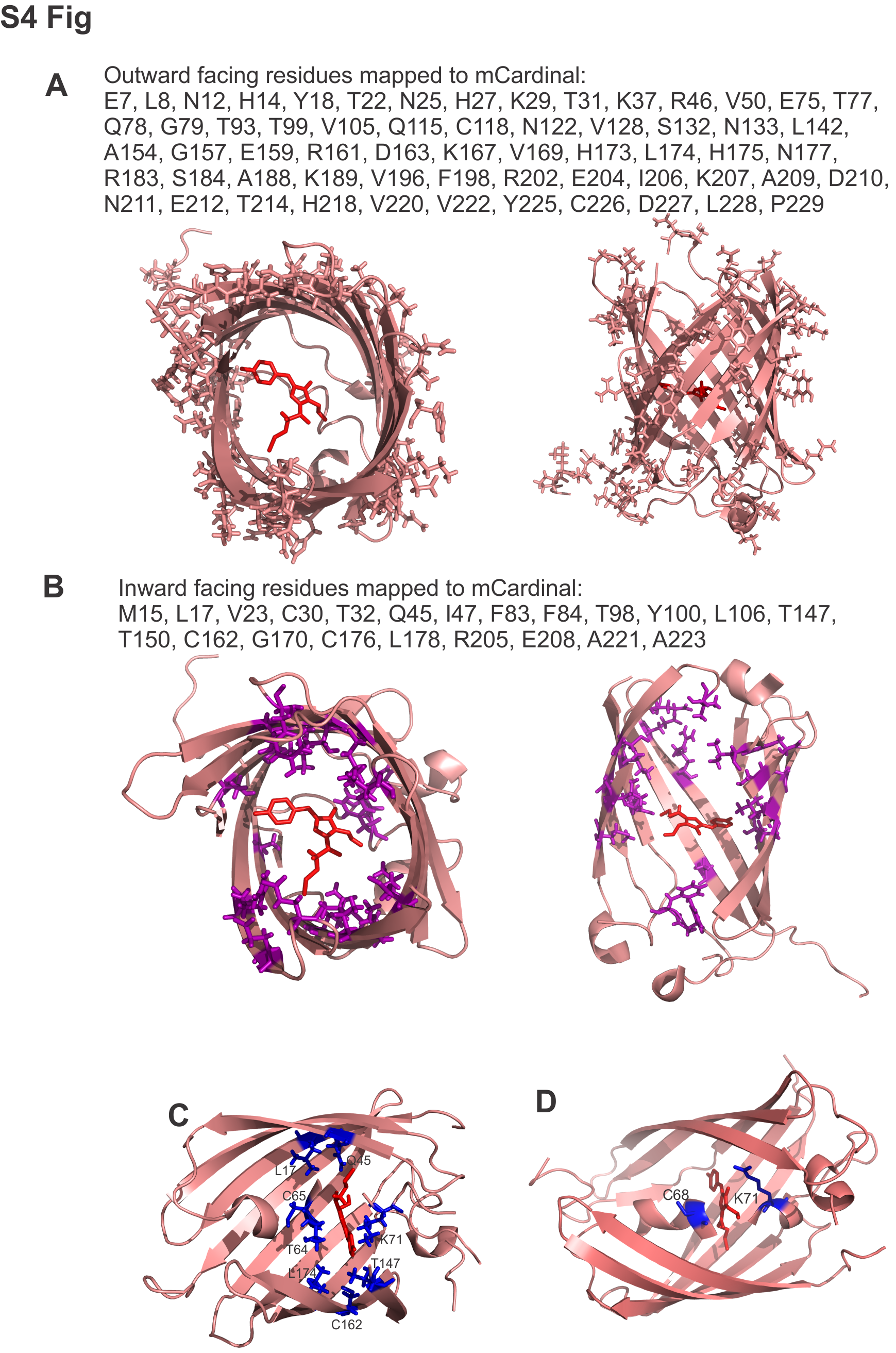

Supplement: S4 Fig — All amino acids that are on the β-strands that facing outward away from the chromophore (A), inward toward the chromophore (B), or the nine closest inward facing amino acids (C). All residues are shown on mCardinal (PDB ID: 4OQW). Note that the residues that are on the inner α-Helix are not shown. D The two amino acids that are from mCardinal and close to the chromophore in SL72. These residues are shown on mScarlet (PDB ID: 5LK4) with the mCardinal residues highlighted because SL72 is more similar to mScarlet than mCardinal. (TIF) [file pone.0208075.s004.tif]
